# Calcium is the switch in the moonlighting dual function of the ligand-activated receptor kinase phytosulfokine receptor 1

**DOI:** 10.1186/s12964-014-0060-z

**Published:** 2014-09-23

**Authors:** Victor Muleya, Janet I Wheeler, Oziniel Ruzvidzo, Lubna Freihat, David T Manallack, Chris Gehring, Helen R Irving

**Affiliations:** Monash Institute of Pharmaceutical Sciences, Monash University, 381 Royal Parade, Parkville, VIC 3052 Australia; Department of Biological Sciences, North-West University, Private Bag X2046, Mmabatho, 2735 South Africa; Division of Biological and Environmental Sciences and Engineering, 4700 King Abdullah University of Science and Technology, 23955-6900 Thuwal, Kingdom of Saudi Arabia

**Keywords:** Calcium, Guanylate cyclase, Kinase, PSKR1

## Abstract

**Background:**

A number of receptor kinases contain guanylate cyclase (GC) catalytic centres encapsulated in the cytosolic kinase domain. A prototypical example is the phytosulfokine receptor 1 (PSKR1) that is involved in regulating growth responses in plants. PSKR1 contains both kinase and GC activities however the underlying mechanisms regulating the dual functions have remained elusive.

**Findings:**

Here, we confirm the dual activity of the cytoplasmic domain of the PSKR1 receptor. We show that mutations within the guanylate cyclase centre modulate the GC activity while not affecting the kinase catalytic activity. Using physiologically relevant Ca^2+^ levels, we demonstrate that its GC activity is enhanced over two-fold by Ca^2+^ in a concentration-dependent manner. Conversely, increasing Ca^2+^ levels inhibits kinase activity up to 500-fold at 100 nM Ca^2+^.

**Conclusions:**

Changes in calcium at physiological levels can regulate the kinase and GC activities of PSKR1. We therefore propose a functional model of how calcium acts as a bimodal switch between kinase and GC activity in PSKR1 that could be relevant to other members of this novel class of ligand-activated receptor kinases.

**Electronic supplementary material:**

The online version of this article (doi:10.1186/s12964-014-0060-z) contains supplementary material, which is available to authorized users.

## Findings

**Figure 1:**
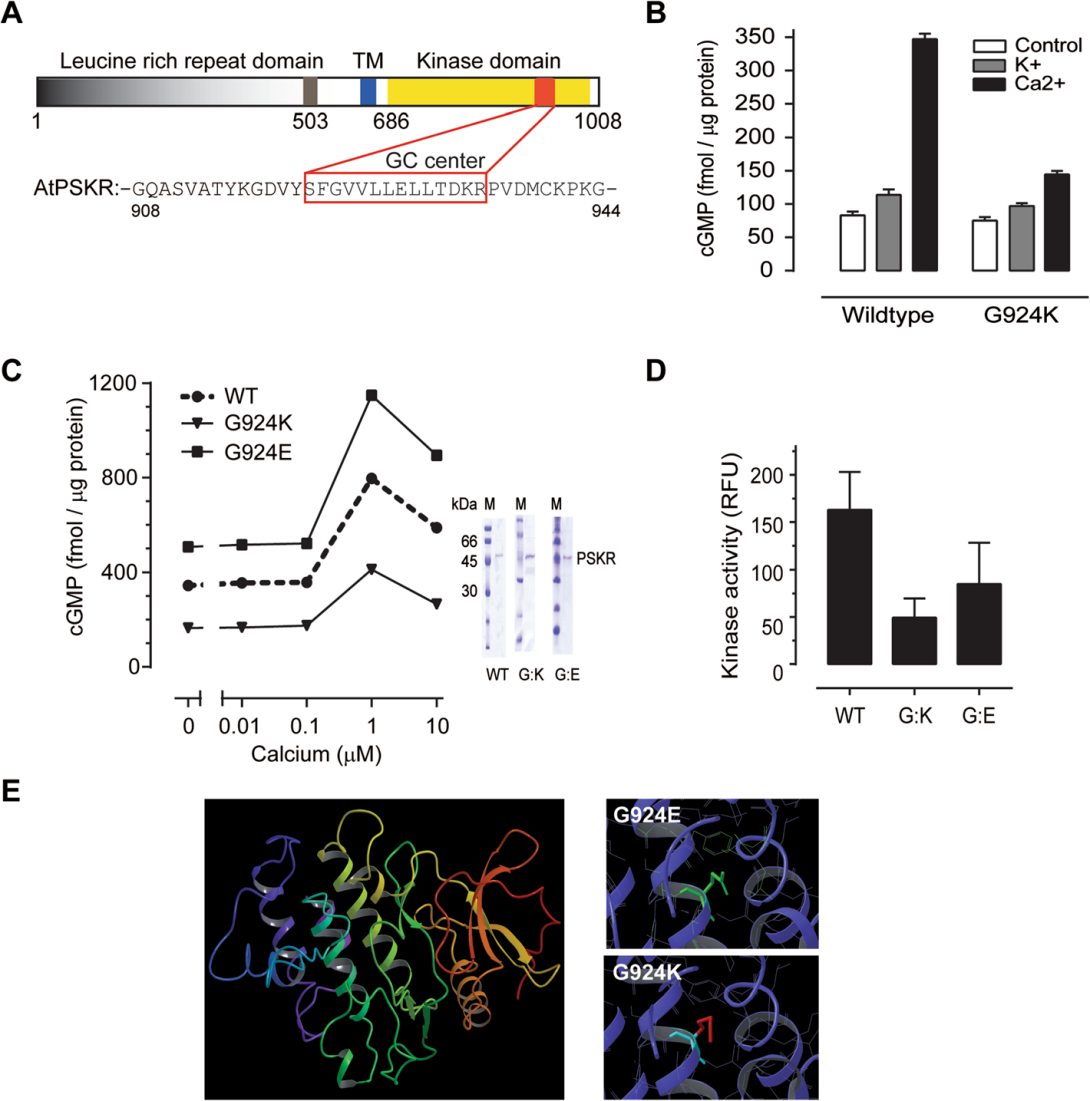
**Effect of calcium on guanylate cyclase activity of PSKR1. A** Schematic diagram of PSKR1 featuring the sequence motif of the guanylate cyclase catalytic centre and the immediately surrounding amino acids (908–944). TM refers to the transmembrane domain and the ligand binding domain occurs in the extracellular region from residues 503 to 517. **B** Effect of cations on guanylate cyclase activity of PSKR1. The cytoplasmic domain of PSKR1 (residues 683 to 1008) was expressed as either wild-type His-tagged SUMO-fused protein or the mutant protein (G924K) prepared as previously described [6]. Calcium significantly enhanced guanylate cyclase activity and the G924K mutant had significantly less activity than the wild-type (mean ± s.e.m., n = 3; *P* < 0.0001 two-way ANOVA, Sidak’s multiple comparison test). **C** The effect of calcium on the guanylate cyclase activity of wild-type and mutant (G924K and G924E) PSKR1 (residues 686 to 1008) was measured at increasing calcium concentrations buffered with EGTA and Mg^2+^. The curves and all the treatments at 1 and 10 μM are significantly different (mean ± s.e.m. (error bars within symbol), n = 3 independent experiments; *P* < 0.0001 two-way ANOVA, Tukey-Kramer multiple comparison test). The purified wild-type and mutant PSKR1 (residues 686–1008) molecules (1 μg) were analysed by SDS-PAGE. **D** The kinase activity of the G924K or G924E mutants (residues 686–1008) determined at 8 nM free calcium were not significantly different to the wild-type (mean ± s.e.m., n = 3 independent experiments; *P* = 0.1532 one-way ANOVA). **E** Homology model of the cytoplasmic domain of PSKR1 was developed based on tomato resistance protein Pto. The homology model was mutated in the guanylate cyclase domain to show the effect of G924E (tolerated) and G924K (steric hindrance). The red colour associated with the lysine residue indicates steric hindrance and strain.

In higher and lower eukaryotes, many receptor kinases contain a putative guanylate cyclase catalytic centre encapsulated in the C-terminal part of the kinase domain (Figure 1A). Candidate receptor kinases with this novel type of overlapping dual-domain architecture are not uncommon since *Arabidopsis thaliana* alone is estimated to have more than 40 members of this new class of proteins [1]. Membrane-bound members of this class of proteins have a typical architecture containing an extracellular ligand binding domain, a single transmembrane spanning domain and an intracellular catalytic kinase domain [2].

In plants, four members of the guanylate cyclase-embedded receptor kinases have been shown to possess a low level of intrinsic guanylate cyclase activity *in vitro;* these are the brassinosteroid receptor (BRI1; BRASSINOSTEROID INSENSITIVE 1) [3], the wall associated kinase-like10 (WAKL10) [4], the elicitor peptide 1 receptor (PepR1) [5] and the phytosulfokine receptor 1 (PSKR1) [6]. All of these molecules have a primary function as kinases and predominantly fold as kinase molecules [1,7]. In another recent study, no guanylate cyclase activity was detected in the BRI1 kinase domain where the assay conditions used favoured kinase activity and the construct lacked the cytoplasmic domains necessary to promote dimerization [7]. Dimerization and/or activation of a molecular switch to turn down the kinase activity may be necessary to generate conformational folding required for guanylate cyclase activity [1].

PSKR1 recognizes the secreted cell proliferation agent, phytosulfokine (PSK), containing sulphated tyrosine residues [8,9] and is essential for cell growth [10-12]. Brassinosteroid signalling enhances PSK expression and PSKR1 dependent quiescent centre cell division [13] and PSK is involved in attenuating stress responses [14] with roles in both immune and developmental processes [15,16]. PSKR1 mediated signalling elicits increases in guanosine 3′,5′-cyclic phosphate (cGMP) in isolated mesophyll protoplasts and transfection of protoplasts with full length PSKR1 results in raised endogenous levels of cGMP [6]. Recently, the kinase activity of PSKR1 has been shown to be essential for PSK signalling *in vivo* [17]. However, the underlying mechanisms regulating the overlapping dual functions of guanylate cyclase-embedded receptor kinases have remained elusive. Here we use PSKR1 as a representative member of this novel class of receptor kinases to unravel the biochemical conditions that enable the dual functions. We show that calcium has opposing effects on the kinase and guanylate cyclase activities of PSKR1. We propose a functional model of how calcium acts as a bimodal molecular switch between these two activities so that they do not occur concurrently.

### Calcium enhances GC activity

**Figure 2:**
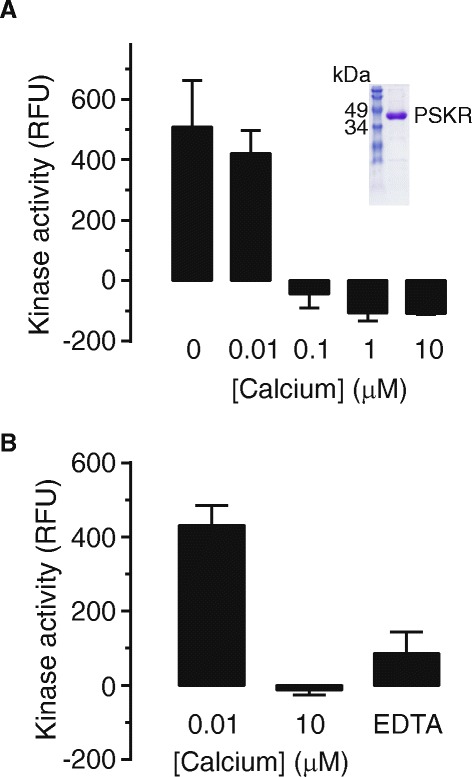
**Effect of calcium on kinase activity of PSKR1. A** Kinase activity (relative fluorescence units (RFU)) of the cytoplasmic domain of wild-type PSKR1 (residues 686–1008) was measured at increasing calcium concentrations buffered with EGTA and Mg^2+^. Kinase activity was significantly reduced at calcium concentrations greater than 0.01 μM (mean ± s.e.m., n = 3 independent experiments; *P* = 0.0008, one-way ANOVA, Tukey-Kramer multiple comparison test). The inset shows the SDS-PAGE analysis of the purified cytoplasmic domain of PSKR1 (5 μg). **B** Suppression of wild-type PSKR1 kinase activity by calcium. Kinase activity of wild-type PSKR1 (residues 686–1008) was determined when incubated in zero calcium and then subjected to 10 μM free calcium before 10 mM EDTA was added. Kinase activity was significantly reduced by the calcium treatment (mean ± s.e.m., n = 3; *P* = 0.0013 one-way ANOVA, Tukey-Kramer multiple comparison test).

When examining the regulation of the cytoplasmic domain of PSKR1 *in vitro,* it was previously found that the kinase activity was inhibited by cGMP [6], suggesting the potential for bimodal modification of the dual activities. Further support for this notion was obtained when the effect of ionic conditions on the cytoplasmic domain of recombinant AtPSKR1 [TAIR:AT2G02220; GenBank:NP_1783300.1] was examined, as guanylate cyclase activity was enhanced in the presence of 5 mM Ca^2+^ but not K^+^ ions (Figure 1B and see Additional file 1: Supplementary Methods). This finding was suggestive that the guanylate cyclase activity of the protein is modulated by a calcium-specific rather than an ion-specific effect. To test the hypothesis that calcium ions modify the enzymatic activities of PSKR1, we used calcium buffer systems to precisely control free calcium ion levels and measured the activities of the cytoplasmic domain of AtPSKR1 (see Additional file 1: Supplementary Methods). Free calcium concentrations were determined using the Maxchelator program taking into account the temperature, pH and ionic strength of the calcium buffer [18]. Guanylate cyclase activity occurred in the absence of calcium and was considerably enhanced at 1 and 10 μM Ca^2+^ but not at lower concentrations in the wild-type protein (Figure 1C). However, the in *vitro* guanylate cyclase activity of PSKR1 is still rather low when compared to canonical membrane bound guanylate cyclases. The G residue in the catalytic motif of GCs is predicted to determine substrate specificity for GTP [19,20] and it was previously shown that when the G residue (924) in the catalytic motif was mutated to K (G924K), it had decreased guanylate cyclase activity [6]. In this study, the G924K mutation conferred reduced guanylate cyclase activity at all calcium concentrations tested however the pattern of response was the same as wild-type protein (Figure 1B and C). Since the positively charged substitution decreased guanylate cyclase activity, we tested the effect of a negatively charged substitution with the expectation that guanylate cyclase activity may be retained. This was indeed the case as the G924E mutant showed enhanced guanylate cyclase activity with an overall similar pattern of response to all physiological calcium concentrations (Figure 1C). Thus increasing free calcium from 100 nM to 1 μM enhanced guanylate cyclase activity by approximately two-fold. Both mutations had no effect on kinase activity when tested at 8 nM Ca^2+^ (one way ANOVA, *P* = 0.1532; Figure 1D) being a calcium concentration favouring kinase activity (see Figure 2A); suggestive that the main function of this region in the kinase domain is to generate cGMP.

We developed a homology model of the kinase domain of PSKR1 (Figure 1E) based on its 41.2 % identity to the crystal structure of tomato resistance protein Pto (for *Pseudomonas syringae* pv tomato) kinase [PDB: 3HGK] [21] (see Additional file 1: Supplementary Methods). When a G924K mutation was incorporated into the model, the molecule became strained due to the steric hindrance encountered by the K residue whereas a negatively charged residue at this position (G924E) was tolerated (Figure 1E) and providing structural reasons for the measured catalytic activities of PSKR1 (Figure 1B and C).

### Calcium suppresses kinase activity

We then determined the effect of precisely controlled calcium levels on the kinase activity of wild-type PSKR1 molecule. The kinase activity of the wild-type protein was completely suppressed at and above 100 nM free calcium (Figure 2A). Kinase activity was rapidly suppressed in response to increased calcium ion concentrations as the same preparation was measured in “zero” calcium where kinase activity was present before being subjected to 10 μM Ca^2+^, resulting in inhibition of the activity. Addition of EDTA to reduce the calcium ion levels resulted in a relatively small increase in kinase activity which did not return to original levels (Figure 2B).

In conclusion, our findings indicate that the kinase activity of PSKR1 is directly inhibited by increases in free calcium ions and importantly, the guanylate cyclase activity is enhanced at similar calcium concentrations, indicative of a reciprocal regulation of the dual functionality of this molecule. The free calcium concentration in the cytoplasm of plant cells is estimated to be in the magnitude of 50 to 100 nM and to increase approximately two to five-fold upon stimulation, depending on particular signature profiles [22]. In plants, calcium regulates a number of protein kinases either directly or indirectly as a means of modulating their biological activity during signal transduction [22-25]. The kinase domain of PSKR1 also contains a calmodulin binding domain that has been shown to interact with all isoforms of calmodulin and is essential for normal growth [17]. Calmodulin is activated by calcium but how calmodulin binding or calcium (this study) directly modulates kinase activity of PSKR1 is currently unknown. Changes in calcium have direct and opposing effects on the alternate intrinsic activities of PSKR1 with the kinase activity being completely suppressed at the concentrations stimulating guanylate cyclase activity. Hence changes in calcium ions act as a direct molecular switch that enables activation of alternate downstream responses.

An important part of any signalling network is the ability to turn down the cascade at appropriate junctures. In bi-functional molecules such as PSKR1, the fact that different intracellular conditions favour one function over the other may allow these molecules to turn on/off their alternate signalling cascades in response to the cellular environment and thereby fine-tune their cognate signalling networks. Furthermore, the initial finding that cGMP, a product of guanylate cyclase activity, inhibits the kinase activity of PSKR1 [6], provides another switch that augments the effect of calcium and thus enabling PSKR1 to shuttle between its alternate signalling networks. The physiological relevance of such switches is supported by recent observations that PSKR1 mediates a switch from growth and development to plant defence responses [15,16]. The fact that the activation of plant defence responses is dependent on changes in both cytosolic calcium and cGMP [26] further substantiates the importance of the PSKR1 switch mediated by changes in intracellular calcium.

## Additional file

Additional file 1
**Supplementary methods.**

## Abbreviations

BRI1: BRASSINOSTEROID INSENSITIVE 1 (brassinosteroid receptor)
cGMP: Guanosine 3′,5′-cyclic phosphate
GC: Guanylate cyclase
PSK: Phytosulfokine
PSKR1: Phytosulfokine receptor 1
