# Supplementary material for: Calcium is the switch in the moonlighting dual function of the ligand-activated receptor kinase phytosulfokine receptor 1
Source: Cell Commun Signal. 2014 Sep 23;12:60. doi: 10.1186/s12964-014-0060-z (PMC4180545; doi:10.1186/s12964-014-0060-z)
Supplement: Additional file 1 — Supplementary methods. [file 12964_2014_60_MOESM1_ESM.pdf]

Additional File 1: Supplementary Methods

Generation of PSKR1 plasmid constructs and recombinant protein expression

The cytoplasmic domain of AtPSKR1 [TAIR:AT2G02220; GenBank:NP_1783300.1] were prepared as described for the constructs PSKR1-KD2 (886-1008) in pDEST17 and PSKR1-KD3 (883-1008) in pET-SUMO [1]. PSKR1-KD3 and its G924K mutant were used in the experiment in Figure 1B and all other experiments involved PSKR1-KD2 constructs. The PSKR1-KD2 mutants (G924E and G924K) were made using a Phusion site-directed mutagenesis kit (New England BioLabs) using the pENTRY-PSKR1-KD2 (with a stop codon) as template and recombined into pDEST17 for protein expression. All clones were sequenced before use to confirm plasmid identity. Expression and purification of recombinant proteins was as described [1].

***In vitro* activity assays**

Kinase and guanylate cyclase activities were measured as previously described [1]. All standard enzyme assays were carried out as independent triplicate experiments. Calcium concentrations were kept at a physiological range of ≈10 nM unless otherwise mentioned. Free calcium concentrations were determined using the Maxchelator program [2] taking into account the temperature, pH and ionic strength of the calcium buffer [3] and included 5 mM Mg^{2+} ions to act as the metal co-factor. However, the metal co-factor was 5 mM Mn^{2+} in the experiment shown in Figure 1B using the PSKR1 pET-SUMO constructs. For guanylate cyclase activity, the amount of cGMP produced by the recombinant proteins was analyzed using either the Amersham cGMP enzyme immunoassay Biotrak (EIA) System (GE Healthcare) or the Sigma cGMP enzyme immunoassay (EIA) system (Sigma-Aldrich) following the standard acetylation protocol described by the supplier.

Model building and minimisation

A homology model of the kinase domain of PSKR1 was developed based on its 41.2% identity to the crystal structure of tomato resistance protein Pto (for *Pseudomonas syringae* pv tomato) kinase [PDB: **3HGK**] [4] using Prime version 3.1 (Maestro version 9.3, Schrödinger, LLC, New York, USA). Minimisation of the model was undertaken using Macromodel version 9.9 (Maestro version 9.3, Schrödinger), employing the PRCG method and the OPLS_2005 force field. A short period of molecular dynamics (300 K, 10 ps) using Macromodel version 9.9 was undertaken to reduce strain followed by further minimisation. A

Ramachandran analysis (PrimeX 1.9, Maestro version 9.3, Schrödinger) revealed that the backbone dihedral angles fell in the expected regions.

Statistical Analysis

Unless otherwise stated, data is expressed as mean ± s.e.m. and analysed using one-way ANOVA followed by Tukey-Kramer multiple comparison test.

References

1. Kwezi L, Ruzvidzo O, Wheeler JI, Govender K, Iacuone S, Thompson PE, Gehring C, Irving HR: **The phytosulfokine (PSK) receptor is capable of guanylate cyclase activity and enabling cyclic GMP-dependant signaling in plants.** *J Biol Chem* 2011, **286:**22580-22588.
2. **Maxchelator program** [http://www-leland.stanford.edu/~cpatton/webmaxc/webmaxcE.htm] (last accessed 13 August 2014).
3. Bers DM, Patton CW, Nuccitelli R: **A practical guide to the preparation of Ca^{2+} buffers.** *Methods Cell Biol* 2010, **99:**1-26.
4. Dong J, Xiao F, Fan F, Gu L, Cang H, Martin GB, Chai J: **Crystal structure of the complex between *Pseudomonas* effector AvrPtoB and the tomato Pto kinase reveals both a shared and a unique interface compared with AvrPto-Pto.** *Plant Cell* 2009, **21:**1846-1859.
